# Maternal and Newborn Characteristics—A Comparison between Healthy and Thrombophilic Pregnancy

**DOI:** 10.3390/life13102082

**Published:** 2023-10-19

**Authors:** Miruna Samfireag, Ovidiu Potre, Cristina Potre, Radu-Dumitru Moleriu, Izabella Petre, Ema Borsi, Teodora Hoinoiu, Ion Petre, Tudor-Alexandru Popoiu, Stela Iurciuc, Andrei Anghel

**Affiliations:** 1 Department of Internal Medicine, Discipline of Clinical Practical Skills, “Victor Babes” University of Medicine and Pharmacy, No. 2 Eftimie Murgu Square, 300041 Timisoara, Romania; samfireag.miruna@umft.ro (M.S.); tstoichitoiu@umft.ro (T.H.); 2 Advanced Cardiology and Hemostaseology Research Center, “Victor Babes” University of Medicine and Pharmacy, No. 2 Eftimie Murgu Square, 300041 Timisoara, Romania; 3 Department of Internal Medicine, Discipline of Hematology, “Victor Babes” University of Medicine and Pharmacy, No. 2 Eftimie Murgu Square, 300041 Timisoara, Romania; potre.cristina@umft.ro (C.P.); borsi.ema@umft.ro (E.B.); 4 Department III of Functional Sciences, Discipline of Medical Informatics and Biostatistics, “Victor Babes” University of Medicine and Pharmacy, No. 2 Eftimie Murgu Square, 300041 Timisoara, Romania; radu.moleriu@umft.ro (R.-D.M.); petre.ion@umft.ro (I.P.); tudor.popoiu@student.umft.ro (T.-A.P.); 5 Faculty of Mathematics and Computer Science, Department of Computer Science, West University of Timisoara, No. 4 Vasile Parvan Boulevard, 300223 Timisoara, Romania; 6 Department XII of Obstetrics and Gynaecology, Discipline III of Obstetrics and Gynaecology, “Victor Babes” University of Medicine and Pharmacy, No. 2 Eftimie Murgu Square, 300041 Timisoara, Romania; petre.izabella@umft.ro; 7 Department VI of Cardiology, Discipline of Internal Medicine and Ambulatory Care, Prevention and Cardiovascular Recovery, “Victor Babes” University of Medicine and Pharmacy, No. 2 Eftimie Murgu Square, 300041 Timisoara, Romania; iurciuc.stela@umft.ro; 8 Department of Biochemistry and Pharmacology, Discipline of Biochemistry, “Victor Babes” University of Medicine and Pharmacy, No. 2 Eftimie Murgu Square, 300041 Timisoara, Romania; biochim@umft.ro

**Keywords:** thrombophilia, pregnancy, newborn

## Abstract

A thrombophilic woman is more likely to experience difficulties during pregnancy, difficulties that will also affect the development of the newborn. This study aims to compare maternal and newborn characteristics between healthy and thrombophilic pregnancy. The following characteristics were analysed: maternal characteristics (BMI- body mass index, haemostasis parameters, thrombophilia-specific treatment) and newborn characteristics (gestational period, birth weight, the Apgar score). This follow-up study spanning five years, from 2018 to 2022, focuses on a cohort of 500 women who underwent delivery hospitalization in the western region of Romania. The maternal characteristics influence the newborn: the greater the weight of the mother with thrombophilia, the more the chances that the fetus will have a lower birth weight; increasing the dose of LMWH (low molecular weight heparin), connected with the necessity to control the homeostasis parameters, the more likely the fetus will be born with a lower birth weight. A pregnant woman with thrombophilia, treated appropriately, having a normal weight, and not presenting other risk factors independent of thrombophilia, will have a newborn with characteristics similar to a healthy pregnant woman.

## 1. Introduction

Hemostasis is a complex and multifaceted process [1]. The process of hemostasis, which the body uses to keep a balance between fluidity and blood coagulation to allow regular blood flow without bleeding, is specific and thoroughly controlled; clinical intervention may be necessary when this balance is perturbed by trauma or underlying bleeding or thrombotic diseases [2].

Molecular abnormalities of hemostasis that predispose to thromboembolic disease or a clinical predisposition to thrombosis are characteristics of thrombophilia [3]. Therefore, thrombophilia can be described as a hereditary or an acquired hemostasis injury that predisposes the affected individuals to venous and/or arterial thrombosis [4]. The condition known as mixed thrombophilia can have both genetic and nongenetic causes. Given that thrombophilia is uncommon in the general population, the presence of genetic or acquired modifications of it is simply one of many factors that define its risk [5].

Numerous changes occur during pregnancy in the process of nourishing the growing fetus and preparing the mother for labor and delivery. While some of these modifications affect metabolic parameters that are normally stable, others could imitate illness symptoms [6]. The hemostatic system undergoes alterations during pregnancy, leading to a hypercoagulable condition, that intensifies as pregnancy progresses and peaks around term [7]. Pregnancy-related changes in the coagulation system result in a physiologically hypercoagulable condition, inducing the risk situations for a possible hemostasis after delivery. Although most patients with hypercoagulability never experience thrombosis, the phrase “hypercoagulable state” is now used interchangeably with “pre-thrombotic state” [4,6].

Thrombotic incidents are now widely acknowledged as a major cause of morbidity and mortality. The susceptibility to clot can be caused by genetic factors, acquired modifications to the clotting process, or, more frequently, a combination of inherited and acquired variables [8]. When choosing the most effective prophylaxis, a pregnant woman with thrombophilia should have been evaluated for the majority of risk factors, also known as triggers for first or recurrent thrombosis [4]. Inherited thrombophilia is the most frequent cause of maternal thromboembolism. This is associated with an increased risk of various adverse pregnancy outcomes, including fetal death in the second and third trimesters, abruptions, severe intrauterine growth restriction, and early-onset, severe preeclampsia [7,9]. Both acquired and hereditary thrombophilia are linked to an increased risk of miscarriage. Maternal thrombophilia is extremely common, particularly in premature placental stillbirth. Stillbirth is a dramatic event that has major psychosocial effects on both the woman and her family. Coagulation abnormalities, both inherited and acquired, should be frequently examined to prevent recurrence [10].

The most prevalent hereditary thrombophilias [11] are related to pathological mutations located at prothrombin (Factor II) gene mutation, in the G20210A position, the factor V Leiden (FVL) gene mutation in the G1691A position [11], the methylenetetrahydrofolate reductase (MTHFR) gene mutation in the C677T and A1298C positions, and the Plasminogen Activator Inhibitor-1 (PAI-1) gene mutation; these pathological mutations can all increase the risk of developing thrombosis. Positive pregnancy outcomes are associated with healthy placental development, a development process that involves a normal fibrinolysis controlled by the coagulation Factor XIII. A pathological mutation, factor XIII Val34Leu may impair fibrinolysis and potentially raise the risk of recurrent pregnancy loss (RPL) in general [12,13,14]. The MTHFR gene pathological mutation is also one of the main risk factors for pregnancy, generated by an affected homeostatic balance [7].

Other factors associated with hereditary thrombophilia are linked with qualitative or quantitative deficiency of protein C, S and antithrombin [15,16]. Protein C and protein S are dependent on Vitamin K and play a crucial role in preserving physiologic hemostasis. Protein C and protein S deficiencies lead to the loss of those organic anticoagulant capabilities, which causes thromboembolism. Reduced plasma antithrombin can be caused by a congenital deficiency or develop secondarily because of several conditions, including liver failure, preterm birth, and sepsis [16,17]. In the presence of documented persistent antiphospholipid antibodies, such as the lupus anticoagulant, moderate-high titer anticardiolipin, or anti-2Glycoprotein I antibodies, the antiphospholipid syndrome is an autoimmune systemic disorder characterized by arterial, venous, or small vessel thrombosis and/or recurrent early pregnancy loss, fetal loss, or pregnancy morbidity [18].

The physiologic changes in the coagulation system, predisposing physical changes like stasis in the large veins of the lower extremities from uterine compression and compression of the left iliac vein by the right iliac artery, decreased mobility, and vascular injury accruing from delivery (especially cesarean birth) all increase the risk of venous thromboembolism (VTE) in all pregnant patients. Traditional VTE risk factors include recent surgery, being older than 35, being obese, smoking, and being sedentary increased risk [19]. There is growing evidence that pregnant women with thrombophilia are more likely to experience various vascular pregnancy problems, such as fetal death, pre-eclampsia, and intrauterine growth restriction (IUGR), in addition to VTE related to pregnancy. Pregnancy can have an impact on a woman’s coagulation and fibrinolytic system. Coagulation tests generally comprise parameters such as prothrombin time (PT), activated partial thromboplastin time (APTT), fibrinogen (FIB), and D-dimers (DD) to evaluate the coagulation system [20].

Biological hypercoagulability is a term used to describe changes in blood coagulation. This is evidenced by increasing concentrations of D-D, the most sensitive sign of secondary fibrinolytic activation [21].

A thrombophilic woman is more likely to experience difficulties during pregnancy. The mixed type of thrombophilia predominates (42.7%) when seen from the perspective of the thrombophilia profile carried out in a study in the western region of Romania [22].

The prothrombotic state of pregnancy makes prothrombotic episodes more noticeable as the gestation progresses. A healthy pregnancy requires the establishment of adequate placental circulation, and inherited thrombophilia can increase the likelihood of unfavorable placenta-mediated pregnancy consequences [23,24,25].

Early pregnancy loss is associated with thrombophilias, posing a significant risk of VTE. There are additional considerations when determining the optimal treatment for pregnant women with thrombophilia, including effectiveness and safety. Clinical decisions about the duration of thrombolytics (such as acetylsalicylic acid) and anticoagulation (such as low molecular weight heparin—LMWH) in pregnant women with thrombophilia are made based on evidence and recommendations [26].

Some suggestions for managing pregnant women with thrombophilia are provided: the extent of genetic damage that presents as thrombotic risk is evaluated during screening. This determines how each patient is treated with anticoagulant and antiaggregant medications. The frequency of patient monitoring will be increased to prevent any thrombotic event that could endanger pregnancy due to the high degree of risk compounded by the development of pregnancy. Women who are prescribed LMWH should get Anti-Xa levels checked more frequently during pregnancy [27].

Antithrombotic precautions will be kept up postpartum, with therapy tailored to the level of risk associated with the severity of thrombophilia [28].

It is crucial to conduct research on how overweight and obese women perform during pregnancy and during delivery in order to improve health outcomes through evidence-based practice. Clinicians struggle to offer sufficient prenatal and birth care for obese women because it requires more expensive medical supplies and equipment [29].

This study aims to compare maternal and newborn characteristics between healthy and thrombophilic pregnancy. The following characteristics were analyzed: maternal characteristics (BMI, hemostasis parameters, the monitoring of the specific treatment that was prescribed in the thrombophilia groups) and newborn characteristics (gestational period, birth weight, and the Apgar score).

Body mass index is a method of categorizing adults based on their height and weight into underweight, normal weight, overweight, and obese categories [30]. The average body mass index has dramatically increased globally, and obesity has been identified as a major public health concern. Type 2 diabetes mellitus, hypertension, coronary heart disease, and stroke are just a few of the health issues for which obesity is a known risk factor [29].

Low birth weight is a strong predictor of early outcomes like development, cognition, and disability and it is defined as less than 2500 g [31]. A low-birth-weight baby is related to a threefold greater incidence of maternal postpartum VTE [32]. Pregnancy, the puerperium, oral contraceptives, and those with the Leiden Factor V mutation have all been linked to an increased risk of thromboembolism. The presence of this mutation is linked to an elevated risk of preterm births and recurrent spontaneous abortions. A 2022 study found a higher prevalence of the Leiden variant among premature neonates with birth weights less than 1500 g [33].

Preterm delivery contributes significantly to infant morbidity and mortality. Although the function of thrombophilia as a risk factor is uncertain, genetic thrombophilia has the potential to cause premature birth [34].

The Apgar scoring method has been used to assess neonatal state and decide whether resuscitation or care escalation, such as admission to a neonatal intensive care unit, is required [35]. The combination of anticoagulant and antiaggregant therapy resulted in a greater gestational age at birth, higher Apgar scores, a higher live birth rate, and a lower abortion rate [36].

## 2. Materials and Methods

This follow-up study spanning five years, from 2018 to 2022, focuses on a cohort of 500 women who underwent delivery hospitalization in the western region of Romania, who were evaluated in hematology and obstetrics and gynecology clinical practice in the western part of Romania. The participants in the study included Caucasian women who were pregnant with a singleton pregnancy at the time of registration, had available results for inherited, acquired, and mixed thrombophilia, had a positive obstetrical history (recurrent pregnancy losses), and underwent LMWH during pregnancy and after delivery.

The thrombophilia screen consisted of the following components: the Factor V Leiden gene mutation, Prothrombin Gene Mutation, methylenetetrahydrofolate reductase (MTHFR) gene mutation, the Plasminogen Activator Inhibitor-1 (PAI-1) gene mutation, the pathological mutation of factor XIII Val34Leu, Antithrombin, Protein C, Protein S, Lupus Anticoagulant, anticardiolipin antibodies, antiphospholipid antibodies, and the homocysteine levels. The exclusion criteria were pregnant women who had not had their regular checkups done, nonpregnant women, subjects with twin pregnancies, and pregnant women who had incomplete results for the thrombophilia screen. After the application of the inclusion and exclusion criteria, the number was reduced to 350 women, who were split into four different groups: 60 patients with hereditary thrombophilia, 60 patients with acquired thrombophilia, 80 patients with mixed (hereditary and acquired) thrombophilia, and 150 healthy pregnant women—the control group.

In addition to documenting the general characteristics of the patients and their newborns, we examined the potential impact of thrombophilia on the baby’s development and pregnancy management.

The World Health Organization’s (WHO) current BMI cut-off criteria for each category is as follows [30]: extremely underweight: lower than 16 kg/m^2^; underweight: 16–18.4 kg/m^2^; normal weight: 18.5–24.9 kg/m^2^; overweight: 25–29.9 kg/m^2^; low-risk obesity: 30–34.4 kg/m^2^; moderate risk obesity: 35–39.9 kg/m^2^; high-risk obesity—a BMI greater than or equal to 40 kg/m^2^.

Preterm babies are those born before 37 weeks of pregnancy. Preterm birth is classified into the following groups, based on gestational age: extremely early (less than 28 weeks), very early (28 to 32 weeks), and moderate to late preterm (32 to 37 weeks) [37].

The Apgar scoring method evaluates the newborn five easily distinguishable components: heart rate, breathing effort, muscular tone, reflex irritation, and color. Each component is assigned a value of 0, 1, or 2, and the total score is the sum of the five component scores. A total score of 7 or more indicates that the baby’s condition is good to outstanding [38].

Several parameters were measured for the patients included in the study: BMI in the first and third trimesters, gestational period (GP), the newborns’ weight, and the APGAR score.

Starting 14 days after the initiation of LMWH, Anti-Xa levels and D-Dimers were tested monthly during pregnancy, in the thrombophilia groups, in order to prevent subsequent problems; in each trimester of the pregnancy, in all of the four groups, hemostatic parameters were checked: PT, INR, APTT, fibrinogen.

Descriptive statistics were conducted, calculating central tendency and dispersion parameters for the numerical variables of the study. For ordinal, nominal, and dichotomous variables, frequency tables were generated, and key percentages were extracted. Data distribution was assessed using the Shapiro—Wilk test. The Mann—Whitney test was employed for comparing two different samples, and the Kruskal—Wallis test was used for more than two different samples. When analyzing the evolution of certain drugs, a Friedman test was employed, and the Wilcoxon Signed Rank test was used to assess differences between two-time points. The study concluded with a linear regression analysis, which included the calculation of Pearson and determination coefficients. The significance level was set at α = 0.05. Microsoft Excel was utilized to compile the database. Two distinct programs were used for statistical analysis: JASPv17.3 and Microsoft Excel.

The database was used with the permission of the Bioethics Commission of Victor Babes University of Medicine and Pharmacy, No. 2 Eftimie Murgu Square, Timisoara, Romania (51/28.09.2018); informed consent was collected from all participants in the study. The study was carried out in accordance with the Helsinki Declaration’s ethical guidelines.

## 3. Results

This follow-up study spanned five years, from 2018 to 2022, involving a total of 350 patients distributed into four groups: 17.14% with hereditary thrombophilia, 17.14% with acquired thrombophilia, 22.86% with mixed (hereditary and acquired) thrombophilia, and 42.86% healthy pregnant women (see Table 1).

Most of the patients were living in an urban environment (276 patients—78.86%).

The lowest newborn weight, gestational period (GP), and APGAR score were in the hereditary thrombophilia group. In almost all the groups, the patients had the same BMI (around 22 kg/m^2^) in the first trimester, slightly higher in the mixed thrombophilia group, yet the highest BMI value was registered in the acquired thrombophilia group.

**Figure 1:**
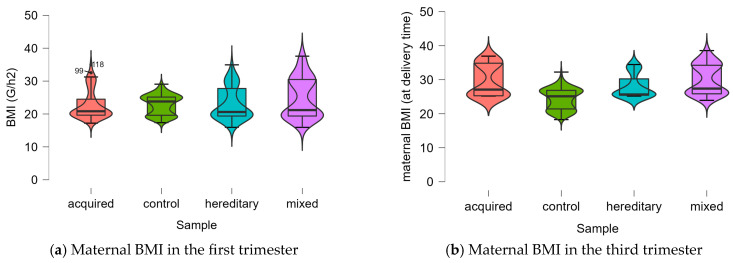

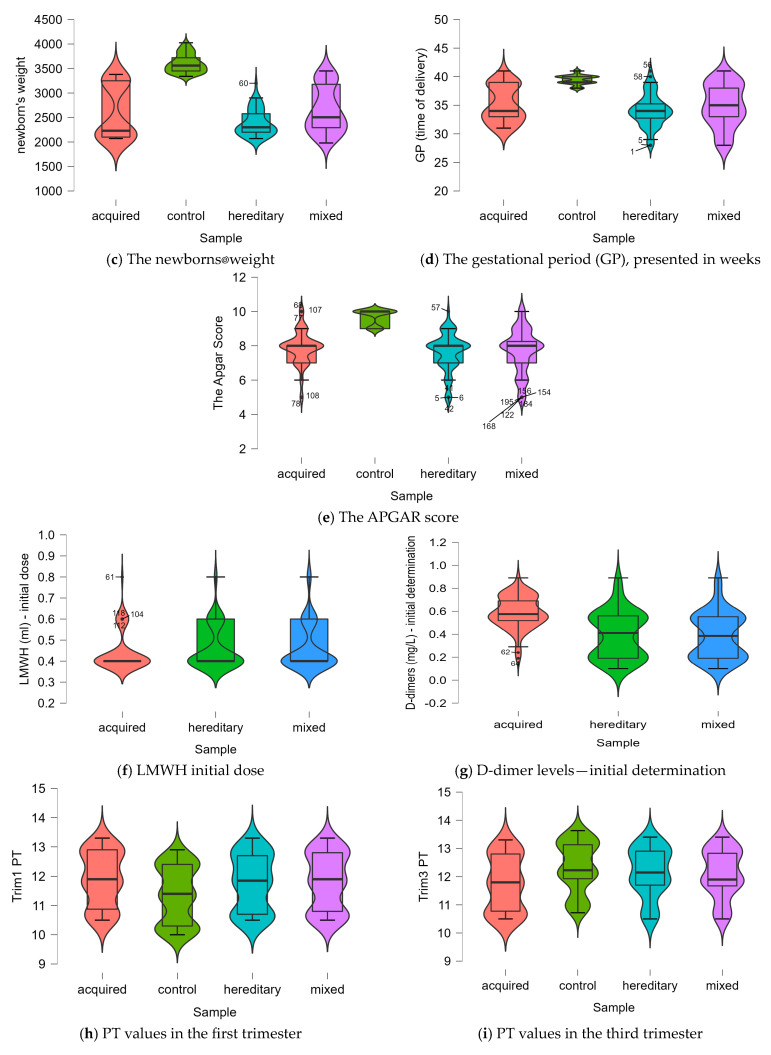

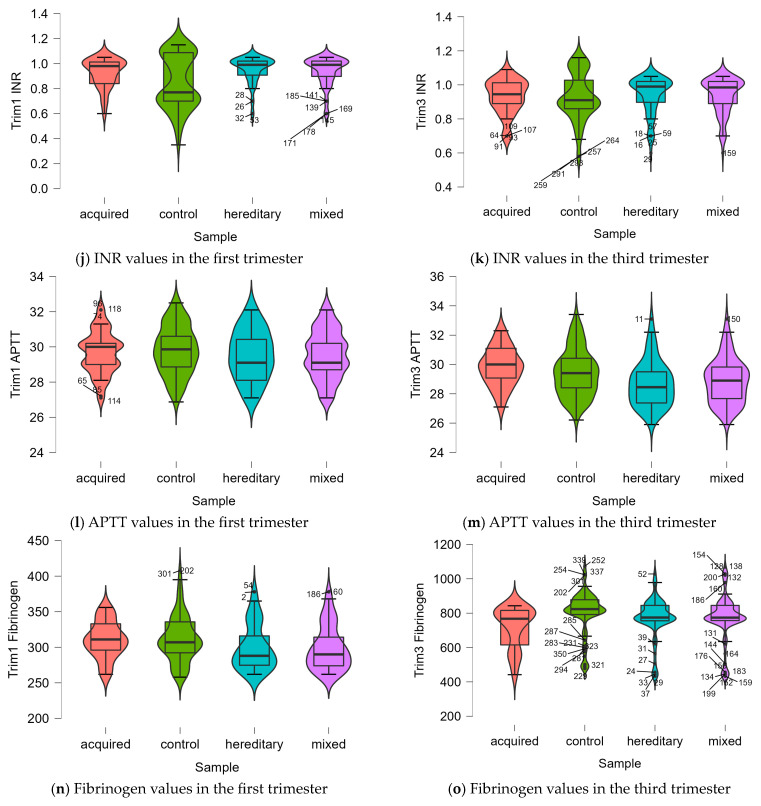
The data distribution of the numerical data, split into the four studied groups.

Using the Shapiro—Wilk test, it was determined that for most of the cases, there was not a normal distribution $\left(p<0.05\right)$, so further on, non-parametrical tests will be used in the statistical analysis. All the results are plotted in Figure 1.

The Mann—Whitney test was applied to compare the control group with each of the thrombophilia groups: with acquired, hereditary, or mixed thrombophilia. In most of the cases, significant differences were obtained $\left(p<0.05\right)$, practically better results were registered in the control group. The Wilcoxon Signed Rank test was applied to see the data evolution between the first and the third trimester, on the evaluated variables, most of the data were revealed as significant (p<0.05). The whole analysis is presented in Table 2 and Table 3.

**Figure 2:**
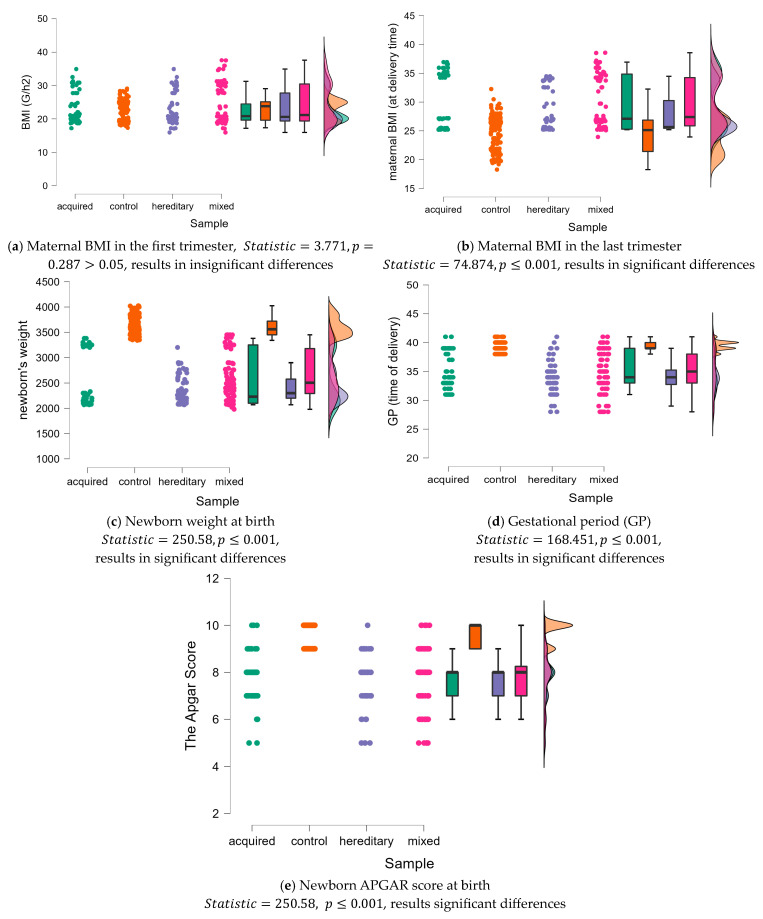
The data dynamics resulted from the Kruskal—Wallis test upon the studied groups for maternal BMI in the first and last trimester, newborn weight, gestational period, and APGAR score. The statistical significance is written below each chart.

The Kruskal—Wallis test was applied to see exactly the data dynamics within the studied groups, and beside the BMI in the first trimester, where insignificant differences were found $\left(p>0.05\right)$, in rest, extremely significant differences were registered $\left(p<0.001\right)$, (see Figure 2).

For the haemostasis parameters and for the doses of LMWH, analysed between the three studied types of thrombophilia, a Kruskal—Wallis test was applied, in each of the nine-time points (one measurement monthly), obtaining insignificant differences, p>0.05, for the LMWH doses and for the D-dimers levels; the same results $\left(p>0.05\right)$, were obtained for PT, INR, APTT, and fibrinogen parameters measured in each pregnancy trimester. So, we can presume no different approach regarding doses of anticoagulant.

**Figure 3:**
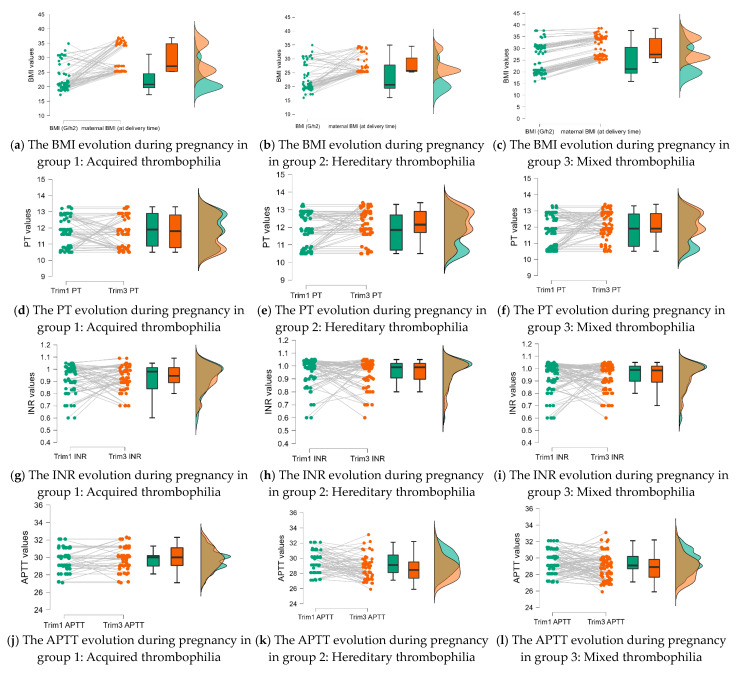

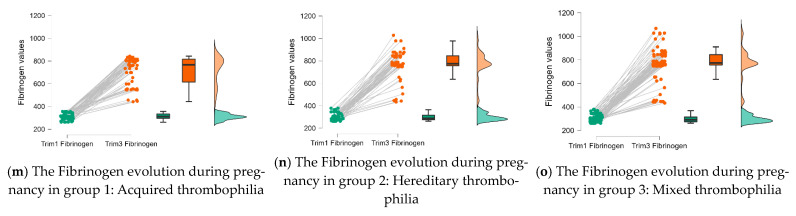
The data distribution was obtained from the Wilcoxon Signed Rank test.

Based on the results from the Mann—Whitney test we applied a Wilcoxon Signed Rank to see the data evolution in the first and last trimester of pregnancy. A significant increase was registered in the case of the mother’s BMI and fibrinogen values, for all three groups of patients with thrombophilia; for hereditary and mixed thrombophilia, it was registered a significant decrease in PT, and in APTT only in the hereditary group (see Table 3, Figure 3).

**Figure 4:**
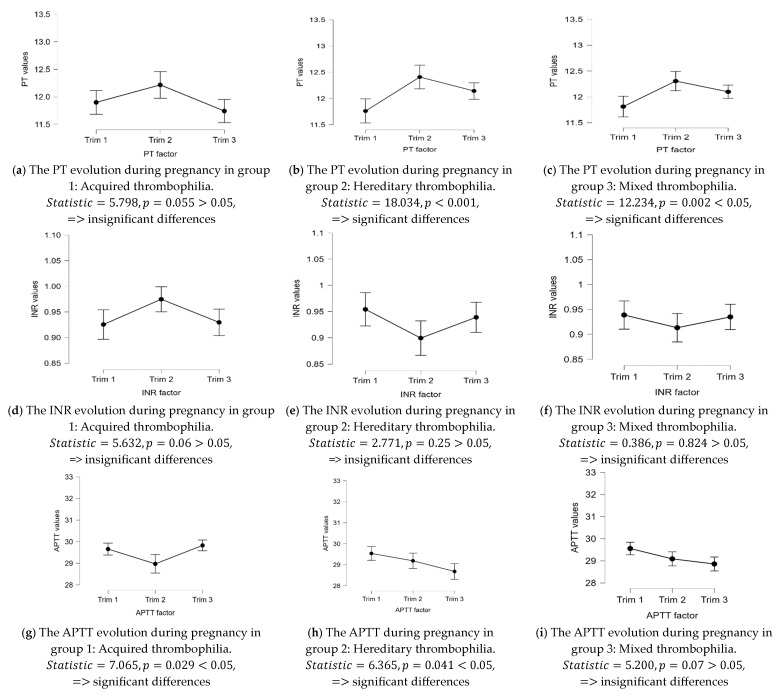

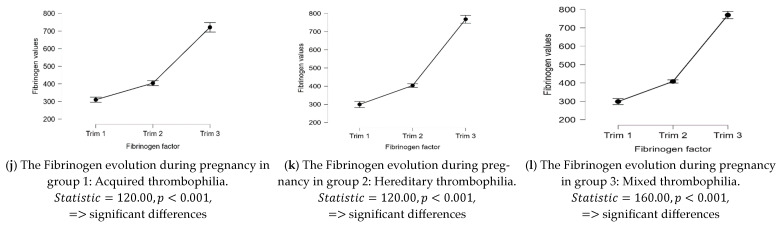
The data distribution for the coagulation parameters (PT, INR, APTT, and Fibrinogen) was obtained from the Friedman test in all three time moments, for all types of thrombophilia, as well as the statistical significance of each case.

For testing the entire evolution of the pregnant women (from the 1st trimester to the 2nd one, and for the 3rd trimester), a Friedman test was applied, obtaining significant differences $\left(p<0.05\right)$ in most of the studied parameters, especially in the hereditary and mixed thrombophilia groups. All the results are presented in Figure 4.

**Figure 5:**
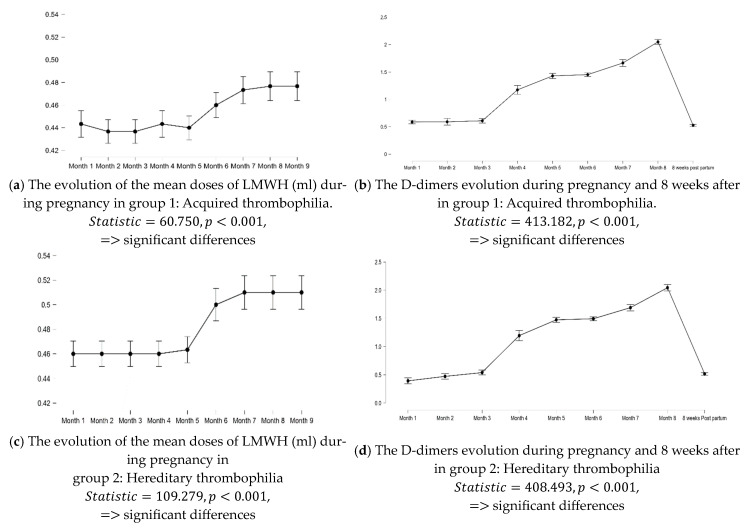

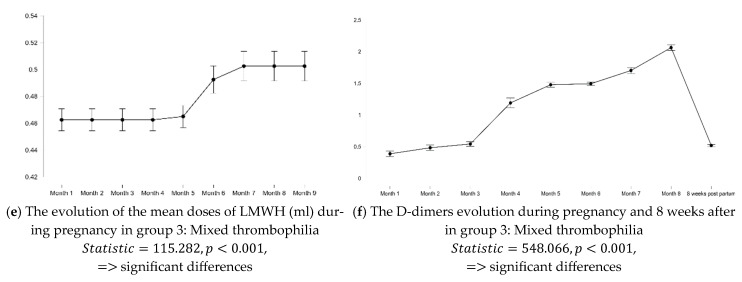
The dose distribution of LMWH (ml), obtained from the Friedman test during pregnancy, for all types of thrombophilia, as well as the statistical significance of each case.

The Friedman test was run to see the mean doses of LMWH (ml) during the 9 months of pregnancy and the mean D-dimers values during pregnancy-time and in the post-partum period, obtaining significant differences $\left(p<0.001\right)$ regarding the administration of LMWH in all three groups (see Figure 5).

**Figure 6:**
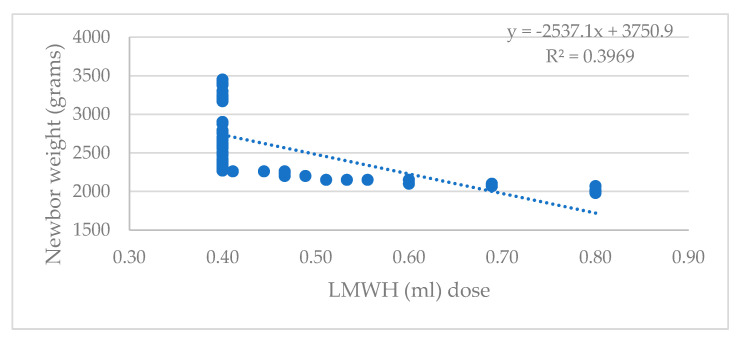
The regression model presenting the dependence between the dose of LMWH and the newborn weight, using a scatter plot chart $\left(r=-0.63;\;R^{2}=39.69,\;p<0.001\right)$.

A regression model was performed to evaluate a possible correlation between the LMWH doses and the newborn weight (see Figure 6).

.

**Figure 7:**
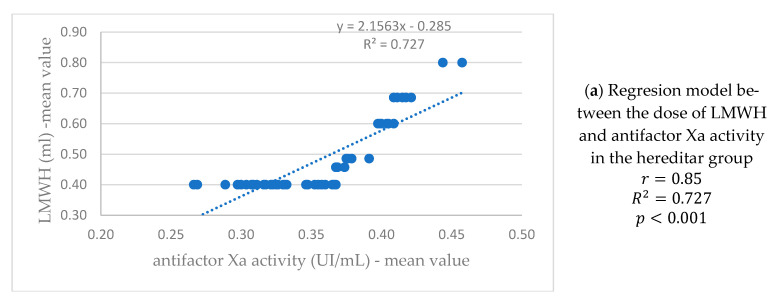

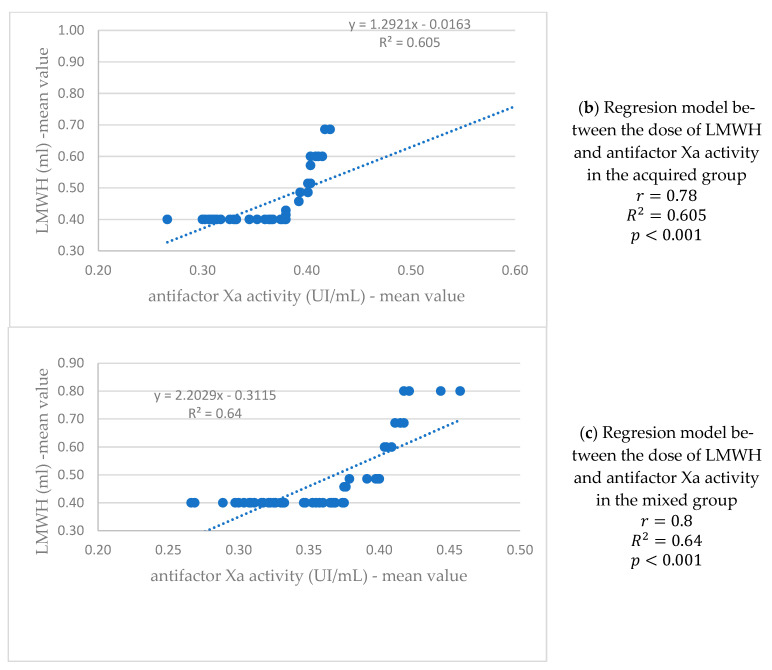
The regression model presents the dependence between the dose of LMWH (ml) and the anti-Factor Xa levels in all three samples.

Another model was performed to test the possible association between the dose of LMWH (ml) and the anti-Factor Xa levels (UI/mL) (see Figure 7).

## 4. Discussion

We conducted a follow-up study spanning five years, from 2018 to 2022, focusing on a cohort of women who underwent delivery hospitalization in the western region of Romania.

Thrombophilia has been associated with both fetal and maternal pregnancy complications, leading to the hypothesis that disruptions in maternal hemostasis may result in reduced fetal growth [39]. Our findings indicate that mothers diagnosed with thrombophilia face a significantly elevated risk of delivering underweight babies, (p<0.05). In our three thrombophilia groups, the mean birth weight of newborns was as follows: 2389.50 g in the hereditary thrombophilia group, 2594.16 g in the acquired thrombophilia group, 2643.00 g in the mixed group, whereas the control group had a mean birth weight of 3598.26 g, aligning with the findings of other research studies [40].

In our study, we discovered a strong link between a good Apgar score and healthy pregnant women (p<0.05): in the control group, the mean Apgar score was 9.71, while in the thrombophilia groups, we observed that the mean Apgar score was 7.60 in the hereditary thrombophilia group, 7,88 in the acquired thrombophilia group and 7.70 in the mixed thrombophilia group. Our findings show that thrombophilic women are considerably more likely to have babies with low Apgar scores.

BMI was found to be significantly related to Apgar scores (p<0.05). Compared to women with normal weight, both overweight and obese women had significantly higher chances of delivering babies with lower Apgar scores (p<0.05) [41]. At enrolment, the mean BMI values were 22.81 in the hereditary thrombophilia group, 22.71 in the acquired thrombophilia group, and 24.88 in the mixed thrombophilia group, while the control group had an average BMI value of 22.81. At delivery, our findings indicated the following average BMI values: 27.96 in the hereditary thrombophilia group, 29.55 in the acquired thrombophilia group, and 30.03 in the mixed thrombophilia group, compared to 24.36 in the control group. Among the 200 participants in the thrombophilia groups, our study revealed that newborns of mothers with a BMI of 25 or higher at the time of delivery had an increased likelihood of having low Apgar scores (p<0.05).

The mean gestational age at delivery in our study was 34.08 weeks in the hereditary thrombophilia group, 35.15 weeks in the acquired thrombophilia group, 34.90 weeks in the mixed group, and 39.43 weeks in the control group. Furthermore, newborns in the research group had lower birth weights, compared to those in the control group.

Hemsworth et al. discovered an elevated risk of small gestational age in pregnancies complicated by FVL mutation in both cohort and case-control study designs in a meta-analysis [42]. This corresponded to the outcomes of our investigation.

In well-resourced countries, VTE is a prominent cause of maternal death [43]. Thrombophilia is a condition characterized by a higher proclivity to VTE [44]. Anticoagulation with low molecular weight heparins is a well-established antithrombotic treatment for thromboprophylaxis during pregnancy, both primary and secondary [45]. LMWH dose modifications during pregnancy based on anti-factor Xa activity levels were common studies of pregnant women on LMWH for VTE prophylaxis. The mean starting dose of LMWH was 0.46 mL in the hereditary and mixed group of thrombophilia, respectively 0.44 mL in the acquired group of thrombophilia. Given our findings and those of other researchers, it seems appropriate to serially evaluate anti-Factor Xa levels in all pregnant women receiving LMWH, because the dosage response to LMWH changes throughout pregnancy. We tested the D-dimers and the anti-factor Xa levels monthly, for all the studied thrombophilia groups. Monitoring the pharmacological action of LMWH is required in certain circumstances (such as pregnant women), where anti-FXa level measurement is advised. In our study, however, the mean anti-FXa levels in pregnant women with thrombophilia were as follows: 0.35 UI/mL in the hereditary thrombophilia group and 0.36 UI/mL in the acquired and mixed thrombophilia groups. In this cohort study of pregnant women who received LMWH for prophylaxis, anti-FXa levels were used to modify LMWH doses during pregnancy; the more we decreased the doses of LMWH, the more anti-FXa levels were within the parameters.

According to a study conducted in 2011 [27] a significant increase in the LMWH dose requirements in the prophylactic group suggests that more frequent monitoring of anti-Factor Xa activity may be appropriate in pregnant patients to maintain target anticoagulant levels.

D-dimer levels rise throughout pregnancy, eventually exceeding the level required to diagnose thromboembolism in the nonpregnant population [46]. We investigated the evolution of D-dimers throughout pregnancy in all the studied thrombophilia groups and found a significant rise in all situations (p<0.05), regardless of the type of thrombophilia our patients possessed.

Prothrombin time (international normalized ratio) or activated partial thromboplastin time has been utilized to identify and evaluate coagulation problem correction or hemostasis [46]. Despite the lack of evidence supporting their accuracy in evaluating the coagulation status of pregnant women, standard coagulation tests are routinely employed. The levels of fibrinogen rise during pregnancy. These levels rise dramatically after the 28th week of pregnancy and are twice as high at the conclusion of the pregnancy [46]. There are now similar studies on pregnant women, to our awareness: in one study, PT and APTT were shown to be substantially shorter, but fibrinogen and D-Dimers plasma concentrations were significantly greater, particularly in the third trimester [20,47], confirming our findings.

A 2010 study indicated no increased risk of low birth weight in mothers who were given LMWH [48]. According to our results, increasing the LMWH (ml) dose causes the newborn’s weight to decrease.

Another risk factor that might affect newborns is the habit of smoking during pregnancy. Smoking has been demonstrated to induce vascular damage. Smoking women are more than twice as likely to have a baby with a low birth weight [49]. Smoking is also an aggravating factor in the evolution of thrombophilia, characterized by an increase in D-dimers along with an increase in treatment requirements [22].

Genetic disorders known as inherited thrombophilias raise the likelihood of developing thromboembolic illness [50]. The hypercoagulable state brought on by typical physiological alterations in various coagulation factors during pregnancy increases the thrombogenic potential of these hereditary diseases. Expanded plasma volume, physiologic anemia, modest neutrophilia in some people, and a slight prothrombotic condition are among the most significant hematologic changes. These predicted physiologic changes must be distinguished from those brought on by pregnancy-related problems by the practitioner [51].

We based our findings on mothers included in the study giving birth in western Romania [50,51]. When determining whether to begin researching the presence of thrombophilia, the age of the female patient, as well as other maternal factors such as recurrent pregnancy losses, BMI, and family history, should be looked over.

## 5. Conclusions

The present study aimed to compare maternal and newborn characteristics between healthy and thrombophilic pregnancy. This study concludes the following regarding the impact of thrombophilia on mothers and their newborns:

- The mixed kind of thrombophilia is the most common (40%).
- The maternal characteristics influence the newborn—according to the Kruskal Wallis test, resulted that the newborn weight is significantly higher in the control group (p<0.05), compared with the thrombophilia groups. A negative correlation was determined between thrombophilia maternal BMI at delivery and the newborns’ weight $\left(r=-0.72,\;R^{2}=0.518,\;p<0.001\right)$, the greater the weight of the mother with thrombophilia, the more the chances that the fetus will have a lower birth weight.
- Increasing the dose of LMWH, the more likely the fetus will be born with a lower birth weight (p<0.05).
- After analyzing the particularities of the homeostatic parameters between the three thrombophilia groups, statistical differences were obtained. The integrity of the pregnant woman’s homeostatic system is also a parameter that influences the characteristics of the newborn; our data suggest that in the case of the women diagnosed with hereditary and mixed thrombophilia, with decreased values for PT and APTT, the weight of the newborn will be lower (p<0.05); also, in all the thrombophilia groups, significant correlations were obtained between the level of maternal fibrinogen and the impact on the development of the newborn (p<0.05).
- A pregnant woman with thrombophilia, treated appropriately and accordingly, having a normal weight, and not presenting other risk factors independent of thrombophilia, will have a newborn with characteristics similar to a healthy pregnant woman.

## Figures and Tables

**Table 1:** Distribution of the studied groups.

Variables	Hereditary Thrombophilia	Acquired Thrombophilia	Mixed Thrombophilia	Control
Percentage	17.14%	17.14%	22.86%	42.86%
Number	60	60	80	150

**Table 2:** The Mann—Whitney test applied to the acquired/hereditary/mixed thrombophilia group compared to the control group.

Statistics	Acquired Thrombophilia vs. Control Group		Hereditary Thrombophilia vs. Control Group		Mixed Thrombophilia vs. Control Group	
Studied Variables	Statistics	*p*-Value	Statistics	*p*-Value	Statistics	*p*-Value
BMI (kg/$m^{2}$)	4069.500	0.280	4862.000	0.363	5214.500	0.102
Maternal BMI (at delivery time)	6738.000	<0.001	2601.000	<0.001	2347.000	<0.001
Newborns’ weight	27.000	<0.001	9000.000	<0.001	11,744.500	<0.001
GP (time of delivery)	1077.000	<0.001	8564.000	<0.001	10,408.500	<0.001
The Apgar Score	483.000	<0.001	8688.500	<0.001	11,195.000	<0.001
1st Trim PT	6089.000	<0.001	3208.000	0.001	4134.000	<0.001
1st Trim INR	5189.500	0.083	3578.500	0.021	4957.000	0.030
1st Trim APTT	4212.000	0.469	4985.000	0.223	6612.000	0.203
1st Trim Fibrinogen	4536.500	0.928	5661.000	0.004	7527.000	0.001
2nd Trim PT	3431.500	0.007	4969.000	0.238	7034.000	0.031
2nd Trim INR	8196.000	<0.001	2370.000	<0.001	2780.000	<0.001
2nd Trim APTT	3303.000	0.003	5416.000	0.021	7443.000	0.003
2nd Trim Fibrinogen	5055.000	0.163	4345.500	0.698	5814.500	0.700
3rd Trim PT	2554.000	<0.001	5404.000	0.023	7399.000	0.004
3rd Trim INR	4710.000	0.598	4028.500	0.236	5525.000	0.323
3rd Trim APTT	5160.000	0.097	5824.000	<0.001	7330.000	0.006
3rd Trim Fibrinogen	2454.000	<0.001	5854.000	<0.001	7824.000	<0.001

**Table 3:** The Wilcoxon Signed Rank test run for the mother BMI, PT, INR, APPT and fibrinogen in all three diseased groups.

Measure 1	Measure 2	Acquired Thrombophilia vs. Control			Hereditary Thrombophilia vs. Control			Mixed Thrombophilia vs. CONTROL		
		Statistics	z-Score	*p*-Value	Statistics	z-Score	*p*-Value	Statistics	z-Score	*p*-Value
BMI (kg/m^2^) 1st Trim	BMI (kg/m^2^) 3rd Trim	154.500	−5.59	<0.001	176.000	−5.44	<0.001	1.000	−7.765	<0.001
1st Trim PT	3rd Trim PT	369.500	1.231	0.221	108.500	−2.73	0.006	277.000	−2.182	0.029
1st Trim INR	3rd Trim INR	867.000	−0.35	0.726	1005.000	0.906	0.367	1704.000	0.606	0.546
1st Trim APTT	3rd Trim APTT	46.000	−1.44	0.155	1352.000	3.217	0.001	2287.500	3.202	0.001
1st Trim Fibrinogen	3rd Trim Fibrinogen	0.000	−6.74	<0.001	0.000	−6.74	<0.001	0.000	−7.77	<0.001

## Data Availability

The use of the database was possible with the agreement of the Bioethics Commission.
